# Inflammatory Markers Mediate the Prognosis of Baseline Mismatch Volume and 90‐Day Outcomes in Acute Ischemic Stroke Patients

**DOI:** 10.1002/brb3.71219

**Published:** 2026-01-19

**Authors:** Lianhong Ji, Xinyu Xu, Peian Liu, Li Li, Jiale Gan, Junqi Liao, Yongxing Deng, Hui Jiang, Yunfei Han, Wenlei Li, Yuan Zhu, Minghua Wu

**Affiliations:** ^1^ Department of Neurology Affiliated Hospital of Nanjing University of Chinese Medicine Nanjing China; ^2^ Department of Neurology Jiangsu Province Hospital of Chinese Medicine Nanjing China

**Keywords:** acute ischemic stroke, CT perfusion, inflammatory markers, mismatch volume, prognosis

## Abstract

**Background:**

While previous studies link larger ischemic penumbra volumes to worse AIS outcomes, clinical results are inconsistent. This study seeks to explore how baseline mismatch volume (a proxy for ischemic penumbra) relates to 90‐day AIS prognosis and the mediating role of inflammatory markers herein.

**Methods:**

This study conducted a retrospective analysis of 473 patients with AIS. Logistic regression and restricted cubic spline (RCS) analysis were used to evaluate the relationship between baseline mismatch volume and poor 90‐day outcomes. The mediating role of inflammatory markers was assessed using the bootstrap method. Participants were divided into four groups based on baseline mismatch volume and outcome to further explore the role of inflammation in ischemic penumbra volume and poor outcome.

**Results:**

Logistic regression analysis showed that baseline mismatch volume was significantly associated with poor outcomes at 90 days (OR = 1.04 [95% CI, 1.01–1.06]; *p* < 0.01), and RCS curve analysis showed a linear relationship between the two (*p* > 0.05). Inflammatory markers partially mediated the relationship between the two (NLR: 22.9%, MLR: 17.5%, PNR: 27.4%). In addition, a small number of patients with large mismatch volumes had good outcomes, while patients with small mismatch volumes had poor outcomes, which was significantly related to the severity of their inflammatory response (*p* < 0.001).

**Conclusion:**

Larger baseline mismatch volume, higher NLR and MLR, and lower PNR were significantly associated with poor outcomes in patients with 90‐day AIS. It is worth noting that in some cases with smaller mismatch volume, patients with high NLR and MLR and low PNR still had poor outcomes. Some patients with low NLR and MLR, and high PNR still experienced favorable outcomes, even with larger mismatch volumes. These findings suggest that AIS prognosis is not solely determined by baseline mismatch volume but is also significantly influenced by systemic inflammatory responses.

## Graphical abstract

AIS prognosis is not solely determined by baseline mismatch volume but is also significantly influenced by systemic inflammatory responses. Larger baseline mismatch volume, higher NLR and MLR, and lower PNR were significantly associated with poor outcomes in patients with 90‐day AIS. But in some cases with smaller mismatch volume, patients with high NLR and MLR and low PNR still had poor outcomes. Some patients with low NLR and MLR, and high PNR still experienced favorable outcomes, even with larger mismatch volumes.

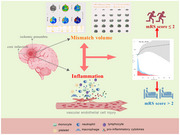

## 1 Introduction

Acute ischemic stroke (AIS) occurs when cerebral tissue experiences hemodynamic changes due to ischemia and hypoxia, leading to insufficient perfusion (Zhu et al. 2022). This can be determined through quantitative cerebral blood flow imaging. When cerebral tissue experiences insufficient perfusion exceeding the injury threshold, irreversible infarction core formation occurs (Heiss and Zaro‐Weber 2019). Baseline infarct core volume can predict clinical outcomes, but individual variations in infarct progression may result in early measurements failing to reflect the disease progression process (Wang et al. 2023). In contrast, the ischemic penumbra volume, influenced by collateral circulation and ischemic time, remains relatively stable. Therefore, using ischemic penumbra volume to predict AIS outcomes over a certain period aligns more closely with the pathophysiological process. With the development of modern imaging technology, CT perfusion (CTP) imaging has emerged as a rapid and convenient method to provide mismatch volume, serving as an alternative quantitative indicator for ischemic penumbra volume (Vinny et al. 2018; Albers et al. 2018).

Mismatch volume is calculated by subtracting core infarction volume from hypoperfused tissue volume and is widely used as a quantitative indicator for the ischemic penumbra (Chung et al. 2023). The ischemic penumbra is the critical region where patients benefit from reperfusion therapy and an important clinical target for the treatment of AIS (Liu et al. 2022, El‐Koussy et al. 2014). Magnetic resonance imaging (MRI) and multimodal CT are the two most commonly used imaging methods. CTP imaging, with its rapid, accurate, and intuitive characteristics, has become an essential method for the initial screening of AIS patients (González 2012; Qiao et al. 2014; Wintermark and Bogousslavsky 2003).

Reperfusion in the ischemic penumbra may trigger oxidative stress and inflammatory cascades, leading to irreversible neuronal damage and impairing post‐AIS neurological functional recovery (Wintermark et al. 2006; Ma et al. 2023). Some studies have suggested that the neutrophil‐to‐lymphocyte ratio (NLR), monocyte‐to‐lymphocyte ratio (MLR), and platelet‐to‐neutrophil ratio (PNR) may serve as potential novel biomarkers involved in the baseline inflammatory process in the ischemic penumbra (Xing et al. 2012; Lux et al. 2020) and influence neurological recovery (Zhang et al. 2025).

During clinical treatment, we found that some AIS patients had a small mismatch volume and a poor 90‐day prognosis, while some AIS patients had a large mismatch volume and a potentially good prognosis. Therefore, this study aimed to explore the relationship between baseline mismatch volume and poor 90‐day outcomes in AIS patients, as well as the potential mediating role of inflammatory markers in the relationship between baseline mismatch volume and poor 90‐day outcomes.

## 2 Methods

### 2.1 Study Design and Participants

**FIGURE 1:**
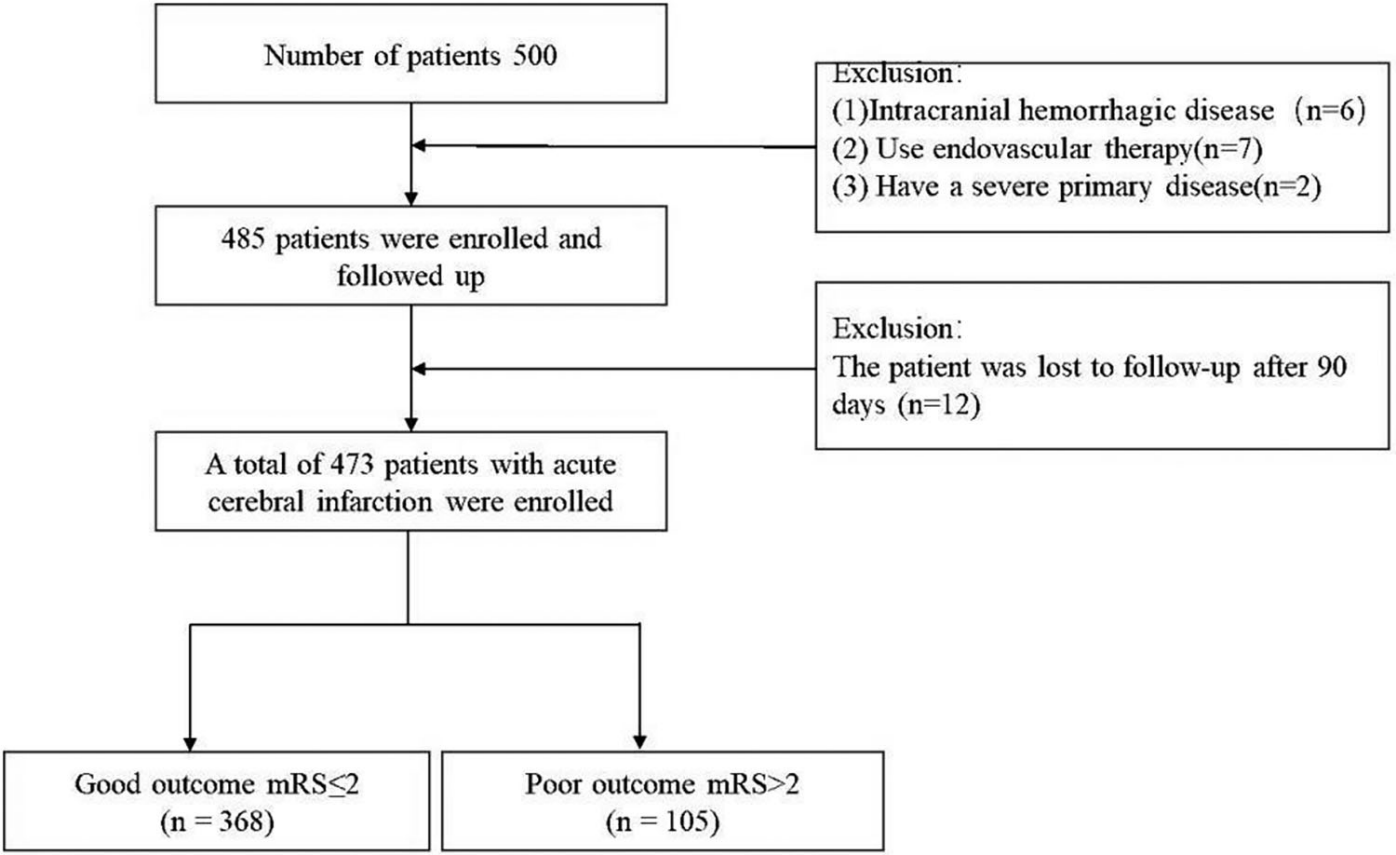
Flowchart for patient selection.

This study included 500 patients with AIS admitted to the Stroke Center between March 2022 and December 2023, with 473 ultimately meeting the inclusion criteria. Inclusion criteria: (1) Age ≥18 years; (2) Diagnosis consistent with the criteria of the Chinese Guidelines for the Diagnosis and Treatment of Acute Ischemic Stroke; (3) Onset within 5 days; (4) Modified Rankin Scale (mRS) score ≤2 before stroke onset; (5) Patients voluntarily signed an informed consent form. Exclusion criteria: (1) Hemorrhagic cerebral infarction or other non‐hemorrhagic intracranial hemorrhagic diseases; (2) Patients who had received thrombolytic therapy or mechanical thrombectomy after onset; (3) Patients with other severe primary systemic diseases (Figure 1).

### 2.2 Data Collection

Demographic characteristics of all AIS patients were collected, including patient age, gender, random blood glucose (RBG), body temperature (BT), cerebrovascular disease risk factors (including hypertension, diabetes, coronary heart disease, hyperlipidemia, atrial fibrillation, and transient ischemic attack (TIA)), smoking and drinking history, medication history (including antiplatelet aggregation drugs, lipid‐lowering drugs, antihypertensive drugs, and antidiabetic drugs), blood pressure at admission, wake‐up stroke, National Institutes of Health Stroke Scale (NIHSS) score, mRS score, post‐stroke seizures, and post‐stroke pneumonia. Venous blood samples were collected within 24 h of admission, and blood data such as neutrophils (N), lymphocytes (L), monocytes (M), and platelets (P) were recorded. NIHSS scores and mRS scales were assessed at 90 days post‐discharge. Prognosis outcomes were determined based on mRS scores. A score of 0 indicates no disability, a score of 2 indicates functional impairment, and higher scores indicate more severe neurological deficits. All patients underwent CTP examination within 24 h of admission.

Medication adherence was assessed using the MMAS‐8 scale. The total score is the sum of scores from 8 questions, ranging from 0 to 8, with higher scores indicating better medication adherence. A score of 7–8 indicates good adherence, 6–7 points indicates moderate adherence, and a score < 6 indicates poor adherence. Rehabilitation adherence was evaluated by the ratio of completed rehabilitation training days to scheduled training days: a ratio < 80% was defined as “poor”, and ≥ 80% as “good”.

### 2.3 Imaging Methods and Related Parameter Calculations

Participants underwent scanning using a Philips Brilliance 128‐slice spiral CT scanner. A contrast agent of 50–70 mL was injected into the elbow vein of the subject at a rate of 5 mL/s. A standard scan was performed from the lower margin of the aortic arch to the cranial apex, with the following parameters: slice thickness：0.625 mm, slice spacing： 0.625 mm, current：250 mA, voltage：120 kV, gantry rotation speed：360°/s, and scan threshold：150 HU. After multiple consecutive scans, the perfusion data were uploaded to an independent workstation. Regions of interest (ROIs) were selected at the level where the lesion was most prominent. The change in CT values caused by the contrast agent reaching the tissue is proportional to the contrast agent concentration at each time point, thereby generating a time‐density curve for each voxel. Parameters such as cerebral blood flow (CBF), cerebral blood volume (CBV), mean transit time (MTT), time to peak (TTP), Tmax, rCBF < 30%, Tmax > 6 s, mismatch volume, and mismatch ratio were calculated. Calculate the relative cerebral blood volume (rCBV) and relative cerebral blood flow (rCBF) in AIS patients. rCBV is the ratio of affected cerebral blood volume to healthy cerebral blood volume (rCBV = affected cerebral blood volume/healthy cerebral blood volume), and rCBF is the ratio of affected cerebral blood flow to healthy cerebral blood flow (rCBF = affected cerebral blood flow/healthy cerebral blood flow). Define the core infarct volume as rCBF < 30%. The mismatch volume is the difference between the hypoperfused tissue volume and the core infarct volume (mismatch volume = Tmax > 6 ‐ rCBF < 30%，unit: mL).

#### 2.3.1 Power Analysis

The primary objective of this study was to investigate the association between baseline mismatch volume and 90‐day poor outcomes (mRS score > 2) in patients with AIS. A multivariate logistic regression design for binary outcomes was adopted, with the significance level (α) set at 0.05 (two‐tailed) and the target power (1‐β) at 80%, consistent with conventional standards in medical research. For the effect size, based on preliminary analysis results and consensus from similar studies, the expected OR for 90‐day poor outcome risk was set at 1.04 per 10 mL increase in baseline mismatch volume. The event rate was determined using data from the 473 actually enrolled patients, among whom 105 (≈22.2%) experienced 90‐day poor outcomes. This rate was highly consistent with the conclusion from the 2023 Chinese Guidelines for the Diagnosis and Treatment of Acute Ischemic Stroke that the 3‐month disability rate in AIS patients ranges from 14.6% to 23.1%. Additionally, through rigorous patient selection and electronic medical record verification, the data completeness rate reached 100%, eliminating the need for additional sample size reservation to account for data loss. Validation using G*Power 3.1 software demonstrated that a minimum of 28 outcome events and an approximate total sample size of 126 were required to detect the core association; the actual sample size and number of events in this study far exceeded these minimum requirements. Reverse calculation showed that under the conditions of α = 0.05 (two‐tailed), OR = 1.04, and 4 independent variables (baseline mismatch volume, NLR, MLR, and PNR), the actual statistical power reached 98.2%. Furthermore, verification using the events per variable (EPV) criterion (EPV = 105 ÷ 4 = 26.25 ≥ 10) confirmed that the multivariate logistic regression model was free from overfitting bias, ensuring the stability and reliability of the estimated regression coefficients.

### 2.4 Statistical Analysis

This study used R statistical software(v 4.3.0) and SPSS (statistical package) version 25.0 for data analysis. Continuous variables were compared using the Whitney test or t‐test, and data were expressed as mean ± standard deviation (mean ± SD). A logistic regression model was used to analyze the relationship between baseline mismatch volume, NLR, MLR, PNR, and poor outcome at 90 days in AIS patients. Spearman's correlation test was used to evaluate the correlation between each indicator and the mRS scale, and the results were expressed as odds ratios (OR) and 95% confidence intervals (CI). Based on the results of univariate analysis and previous literature reports, confounding factors were screened and corrected, and their association with 90‐day poor outcomes in AIS patients was further evaluated through restricted cubic spline (RCS) analysis. At the same time, a forest distribution map was drawn based on subgroup analysis. The bootstrap method was used to test the mediating role of NLR, MLR, and PNR in the relationship between baseline mismatch volume and 90‐day poor outcomes in AIS patients. All tests were two‐sided, and *p* < 0.05 was considered statistically significant.

## 3 Result

### 3.1 Baseline Information

Among 500 patients with AIS, 6 were excluded due to concomitant intracranial hemorrhagic disease, 7 underwent endovascular treatment, 2 had severe primary disease, and 12 were lost to follow‐up at 90 days. Ultimately, 473 AIS patients were included in this study. The mean age of participants was 68.0 ± 11.4 years, with 360 (76.1%) being male. The baseline characteristics of patients in the good outcome group and poor outcome group are shown in Table 1. Compared with the good outcome group, patients in the poor outcome group were older and had a higher baseline mismatch volume, along with higher RBG levels on admission, higher NIHSS scores on admission, and higher mRS scores on admission. Meanwhile, this group had a lower mean diastolic blood pressure on admission, lower usage rates of antiplatelet aggregation drugs and lipid‐lowering drugs, and a higher incidence of post‐stroke epilepsy and pneumonia complications, as well as poorer medication adherence and rehabilitation adherence (*p* < 0.05).

**TABLE 1:** The clinical and biochemical characteristics of the two groups were analyzed.

	Total (n = 473)	Good outcome (n = 368)	Poor outcome (n = 105)	*p*‐value
Male n (%)	360 (76.1)	283 (76.9)	77 (73.3)	0.449
Age, y mean (SD)	68.0 ± 11.4	67.2 ± 11.1	70.7 ± 11.8	0.006
BMI, kg/m^2^ mean (SD)	24.2 ± 2.8	24.2 ± 2.9	23.7 ± 2.0	0.369
Current smoker, n (%)	167 (35.3)	129 (35.1)	38 (36.2)	0.83
Heavy drinker, n (%)	128 (27.1)	98 (26.6)	30 (28.6)	0.693
Admission RBG	7.74±3.98	7.43±3.71	8.8±4.71	0.002
Admission BT	36.4±0.24	36.4±0.23	36.4±0.27	0.197
TG, mean (SD)	4.3 ± 1.2	4.3 ± 1.2	4.1 ± 1.1	0.24
TC, mean (SD)	1.6 ± 1.5	1.6 ± 1.2	1.5 ± 2.4	0.586
HDL, mean (SD)	1.2 ± 0.3	1.2 ± 0.3	1.2 ± 0.3	0.544
LDL, mean (SD)	2.5 ± 0.9	2.5 ± 0.9	2.4 ± 0.9	0.305
Mismatch volume, mean (SD)	55.6±93.9	46.6±77.3	87.2±132.6	< 0.001
Admission SBP, mean (SD)	142.0 ± 23.0	142.9 ± 23.2	138.7 ± 22.4	0.099
Admission DBP, mean (SD)	83.1 ± 13.3	83.9 ± 13.2	80.4 ± 13.2	0.014
Admission NIHSS, mean (SD)	4.9 ± 5.5	3.7 ± 3.7	9.3 ± 7.9	< 0.001
Admission mRS, mean (SD)	2.6 ± 1.5	2.2 ± 1.4	3.8 ± 1.2	< 0.001
Wake‐up stroke, n (%)	90 (19.03)	66 (17.93)	24 (22.86)	0.258
Disease history (%)				
TIA, n (%)	35 (7.40)	27 (7.34)	8 (7.62)	0.923
Stroke, n (%)	142 (30.0)	110 (29.9)	32 (30.5)	0.908
Hypertension, n (%)	365 (77.2)	290 (78.8)	75 (71.4)	0.112
Diabetes mellitus, n (%)	186 (39.3)	148 (40.2)	38 (36.2)	0.456
Coronary heart disease, n (%)	50 (10.6)	38 (10.3)	12 (11.4)	0.746
Hyperlipidemia, n (%)	32 (6.8)	26 (7.1)	6 (5.7)	0.627
Other valvular diseases, n (%)	59 (12.5)	41 (11.1)	18 (17.1)	0.101
Current smoker, n (%)	167 (35.3)	129 (35.1)	38 (36.2)	0.83
Heavy drinker, n (%)	128 (27.1)	98 (26.6)	30 (28.6)	0.693
TOAST subtypes, n (%)	0.105			
Large artery atherosclerosis	342 (72.3)	258 (70.1)	84 (80)	
Cardioembolism	37 (7.8)	28 (7.6)	9 (8.6)	
Small artery	83 (17.5)	73 (19.8)	10 (9.5)	
Other/undetermined	8 (1.7)	6 (1.6)	2 (1.9)	
Urinary infection	3 (0.6)	3 (0.8)	0 (0)	
Medicine use during hospitalization (%)				
Antiplatelet, n (%)	399 (84.3)	322 (87.5)	77 (73.3)	< 0.001
Anticoagulant, n (%)	91 (19.3)	65 (17.7)	26 (24.8)	0.106
Antihypertensive, n (%)	290 (61.2)	227 (61.6)	63 (60)	0.893
Antidiabetic, n (%)	161 (34.1)	132 (36)	29 (27.6)	0.112
Lipid‐lowering, n (%)	421 (89.2)	333 (90.7)	88 (83.8)	0.044
Clinical complications				
Post‐stroke seizures, n (%)	6 (1.25)	3 (0.82)	3 (2.86)	0.1
Post‐stroke pneumonia, n (%)	95 (20.08)	49 (13.32)	46 (43.81)	< 0.001
Diagnostic and therapeutic adherence				
Medication adherence, n (%)	400 (84.57)	320 (86.96)	80 (76.19)	0.002
Rehabilitation adherence, n (%)	7.37 (1.32)	7.47 (0.94)	7.01 (2.14)	0.007

Abbreviations: BMI, body mass index; BT, body temperature; DBP, diastolic blood pressure; HDL, high‐density lipoprotein; LDL, low‐density lipoprotein; mRS, modified Rankin Scale; NIHSS, National Institute of Health Stroke Scale; RBG, random blood glucose; SBP, systolic blood pressure; SD, standard deviation; TC, total cholesterol; TG, triglyceride; TIA, transient ischemic attack; TOAST, Trial of ORG 10172 in acute stroke treatment.

### 3.2 Regression Analysis, Restricted Cubic Spline (RCS) Analysis, and Subgroup Analysis of the Relationship Between Baseline Mismatch Volume and Poor 90‐Day Outcome in AIS Patients

**FIGURE 2:**
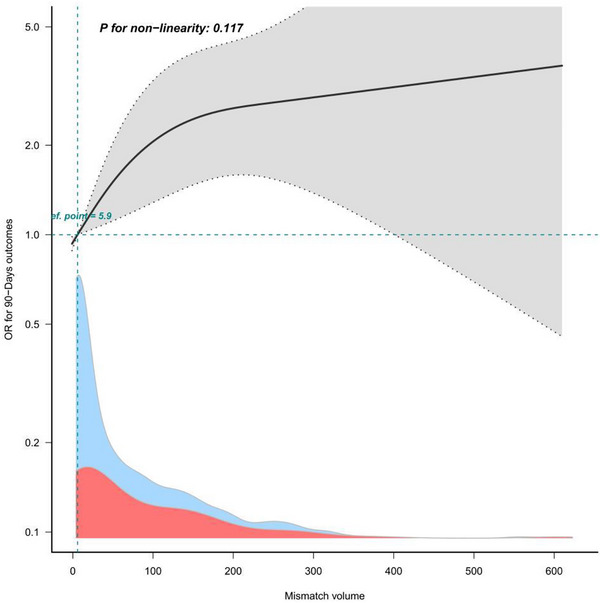
Restricted cubic splines of baseline mismatch volume and 90‐day poor outcomes after AIS.

**FIGURE 3:**
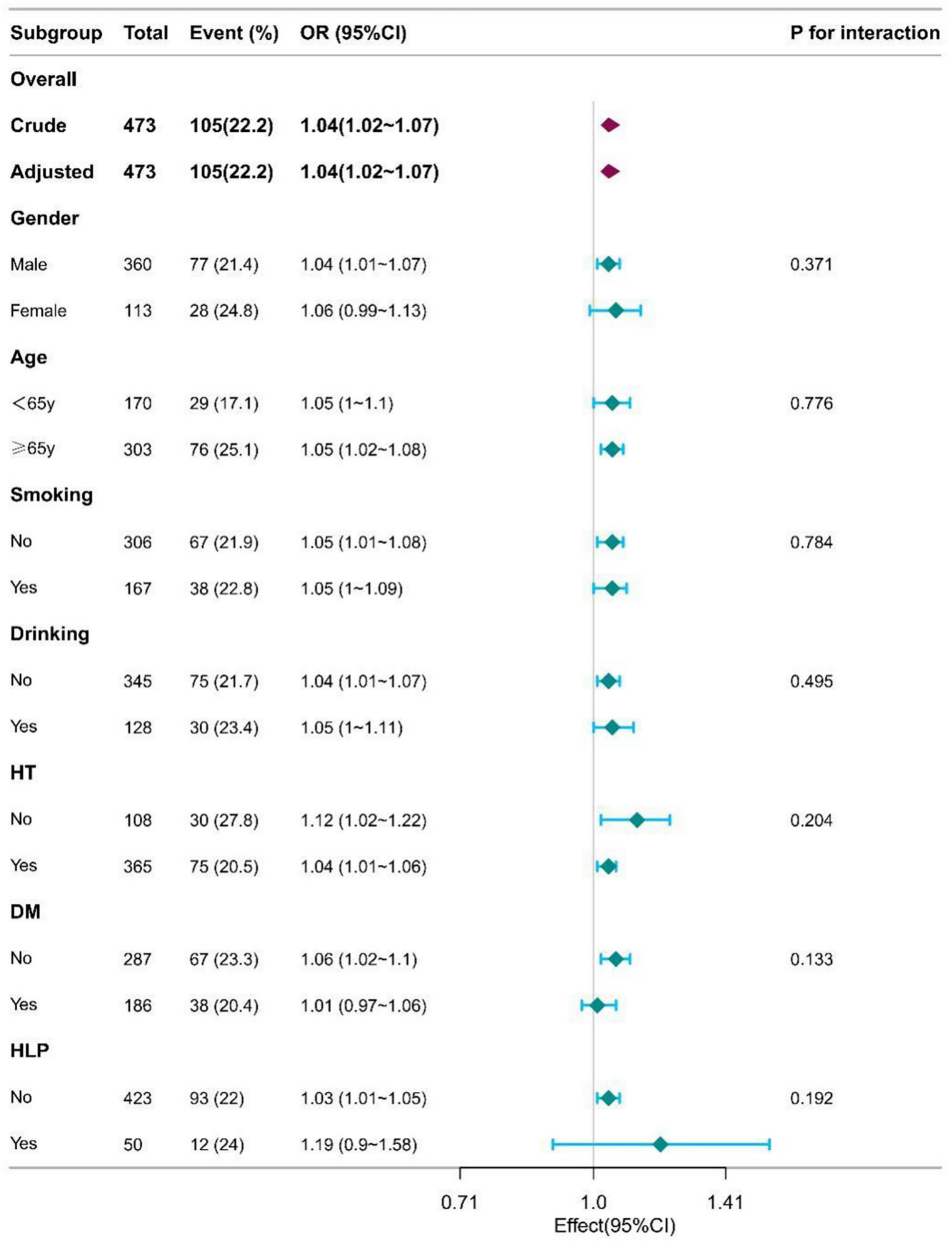
Forest plot of the standard deviation of baseline mismatch volume and poor outcomes by subgroup and interactions. Abbreviations: OR, odds ratio; CI, confidence interval; HT, hypertension; DM, diabetes mellitus; and HLP, hyperlipidaemia.

In the logistic regression analysis, baseline mismatch volume was significantly associated with 90‐day poor outcome in AIS patients (OR = 1.04 [95% CI, 1.02–1.07], *p* < 0.001). After adjusting for gender and age in model 2, the relationship between baseline mismatch volume and 90‐day poor outcome in AIS patients remained statistically significant (OR = 1.04 [95% CI, 1.02–1.07], *p* < 0.001). After adjusting for gender, age, medical history, previous medication history, medication adherence, and rehabilitation adherence in Model 3, the association between baseline mismatch volume and 90‐day poor outcomes in AIS patients remained statistically significant (OR = 1.04 [95% confidence interval, 1.01–1.06], *p* < 0.01) (Table 2). To further explore the relationship between baseline mismatch volume and 90‐day poor outcomes in AIS patients, we adjusted for various potential confounders and performed a RCS analysis, which showed a linear positive correlation between baseline mismatch volume and 90‐day poor outcomes in AIS patients (Figure 2). AIS patients were stratified into different subgroups based on age, gender, smoking history, drinking history, hypertension, diabetes, and coronary artery disease. Subgroup analysis showed that the influence of different subgroups on the relationship between baseline mismatch volume and 90‐day poor outcomes in AIS patients was consistent, with no significant differences between groups (Figure 3).

**TABLE 2:** Univariate and multivariate logistic regression analyses of baseline mismatch volume and 90‐day poor outcomes in AIS patients.

Variable	n total	n. event_%	crude OR (95% CI)	crude. *P* _value	adj. OR (95% CI)	adj. *P*_ value
Model 1	473	105 (22.2)	1.04 (1.02∼1.07)	< 0.001		
Model 2	473	105 (22.2)	1.04 (1.02∼1.07)	< 0.001	1.04 (1.02∼1.07)	< 0.001
Model 3	473	105 (22.2)	1.04 (1.02∼1.07)	< 0.001	1.04 (1.01∼1.06)	0.017

Model 1 was a univariate analysis. Model 2 was adjusted for gender and age. Model 3 was adjusted for gender, age, previous medical history, *Note*: medication history and post‐stroke seizures, and post‐stroke pneumonia. OR: odds ratio; CI: confidence interval.

### 3.3 Regression Analysis and RCS Analysis of the Relationship Between NLR, MLR, and PNR and 90‐Day Poor Outcomes in AIS Patients

In the logistic regression analysis, NLR, MLR, and PNR were significantly associated with 90‐day poor outcomes in AIS patients (OR = 1.21, 1.73, 0.98; *p* < 0.01). After adjusting for gender and age in Model 2, NLR, MLR, and PNR remained significantly associated with 90‐day poor outcomes in AIS patients (OR = 1.21, 1.68, 0.98; *p* < 0.01); after adjusting for gender, age, past medical history, and past medication history in Model 3, the association between NLR, MLR, and PNR and 90‐day poor outcomes in AIS patients remained significant (OR = 1.22, 1.71, 0.98; *p* < 0.01) (Additional file 1: Table S1). To further explore the relationship between inflammatory markers and 90‐day poor outcomes in AIS patients, we included NLR, MLR, and PNR in the RCS analysis. The results showed that NLR and MLR were significantly linearly associated with 90‐day poor outcomes in AIS patients (*p* > 0.05) (Additional file 1: Figures S1 and S2). However, PNR was nonlinearly associated with 90‐day poor outcomes in AIS patients (*p* < 0.05). According to the inflection point analysis, when PNR ≥ 81.34 (i.e., on the right side of the inflection point), PNR was negatively correlated with 90‐day prognosis in AIS patients. Conversely, a positive correlation was observed on the left side of the inflection point. Therefore, high NLR, MLR, and low PNR were positively correlated with 90‐day poor prognosis in AIS patients (Additional file 1: Figure S3).

### 3.4 Mediation Analysis

**FIGURE 4:**
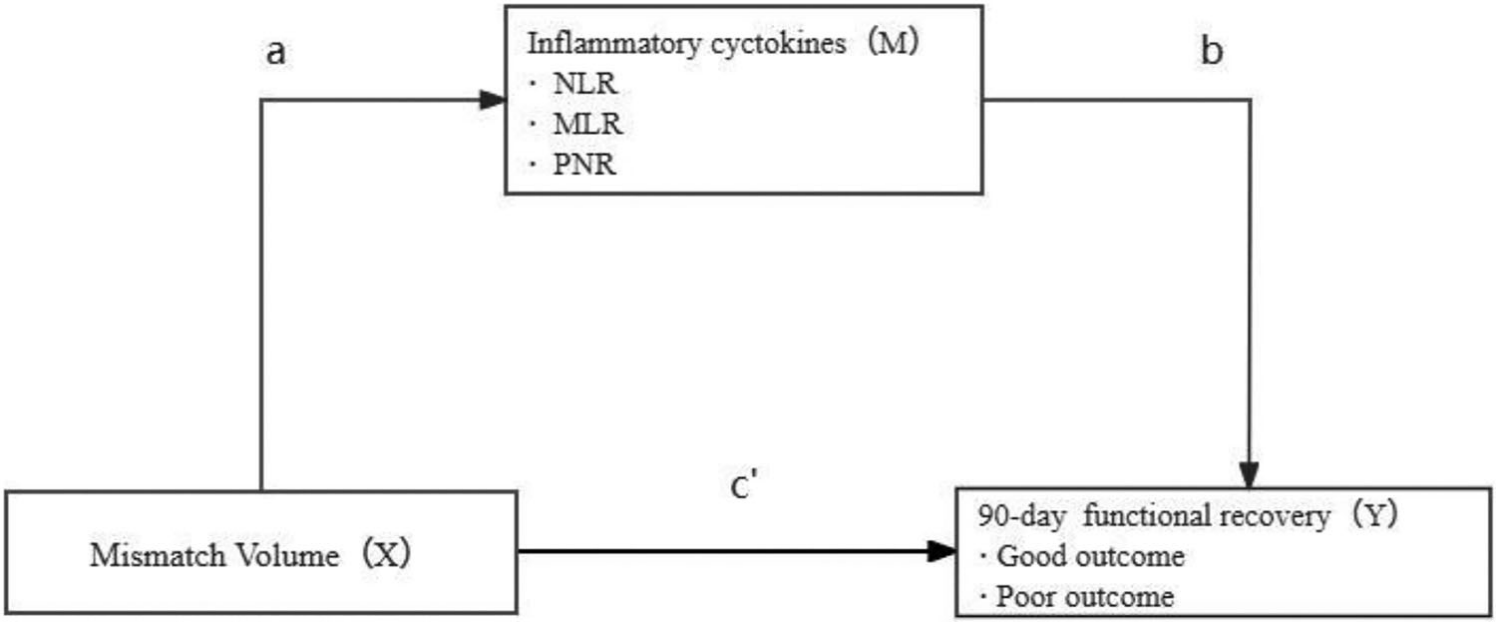
Model of the hypothetical causal pathway in patients with AIS.

Mediator analysis results showed that NLR, MLR, and PNR partially mediated the relationship between baseline mismatch volume and 90‐day poor outcomes in AIS patients (Figure 4), with mediation effects of 22.9%, 17.5%, and 27.4%, respectively (Table 3).

**TABLE 3:** Inflammatory markers have a significant partial mediating effect on the association between baseline mismatch volume and 90‐day poor outcomes after AIS.

Inflammatory markers	Natural direct effect	*p* value	Natural indirect effect	*p* value	Medication effect (%)
NLR	0.0046 (0.0011−0.0077)	0.015	0.0014 (0.0006−0.0029)	< 0.001	22.9
MLR	0.0052 (0.0018–0.0082)	0.004	0.0011 (0.0003−0.0024)	0.0032	17.5
PNR	0.0045 (0.0011–0.0077)	0.013	0.0017 (0.0006−0.0032)	0.0008	27.4

Abbreviations: MLR, monocyte‐to‐lymphocyte ratio; NLR, neutrophil‐to‐lymphocyte ratio; PNR, platelet neutrophil ratio.

To further investigate the impact of inflammatory markers on the association between baseline mismatch volume and 90‐day poor outcomes, patients were divided into low mismatch volume and high mismatch volume groups based on the median baseline mismatch volume (median = 5.9): low mismatch volume < 5.9 versus high mismatch volume ≥ 5.9. Based on the dichotomization of mismatch volume and outcome, patients were divided into four groups: low mismatch volume with good outcome, low mismatch volume with poor outcome, high mismatch volume with good outcome, and high mismatch volume with poor outcome. The study found that in most populations, larger acute cerebral infarction volumes were associated with more severe inflammatory responses and poorer outcomes, while smaller infarction volumes were associated with milder inflammatory responses and better outcomes. However, we also found that a small proportion of patients with large infarction volumes had good outcomes, while some patients with small infarction volumes had poor outcomes. This discrepancy was closely related to the severity of the inflammatory response (*p* < 0.001) (Table 4).

**TABLE 4:** Influence of inflammatory markers on poor outcome at 90 days in patients with different baseline mismatch volumes.

Variables	Total (n = 473)	Group 1 (n = 200)	Group 2 (n = 35)	Group 3 (n = 168)	Group 4 (n = 70)	*P*
NLR, Mean±SD	3.8 ± 3.3	2.9 ± 2.5	4.4 ± 5.0	3.7 ± 2.6	6.3 ± 4.5	< 0.001
MLR, Mean±SD	0.4 ± 0.2	0.3 ± 0.1	0.4 ± 0.2	0.4 ± 0.2	0.5 ± 0.3	< 0.001
PNR, Mean±SD	46.1 ± 25.6	53.9 ± 25.5	45.2 ± 22.9	42.5 ± 23.8	33.0 ± 24.6	< 0.001
*p*		< 0.001		< 0.001		

*Note*: Group 1 was the low mismatch volume with good prognosis group, Group 2 was the low mismatch volume with poor prognosis group, Group 3 was the high mismatch volume with good prognosis group, and Group 4 was the high mismatch volume with poor prognosis group.

## 4 Discussion

The degree of brain tissue perfusion abnormality in the acute phase varies with individual differences (Seners et al. 2022). In this study, baseline mismatch volume was found to be significantly associated with 90‐day poor outcome in AIS patients (i.e., the larger the baseline mismatch volume in AIS patients, the worse the 90‐day prognosis), which may be related to the long‐term lack of reperfusion in ischemic brain tissue and the conversion of some ischemic semidarkened zone tissue into infarct core tissue. Mediation analysis showed that NLR, MLR, and PNR partially mediated the relationship between baseline mismatched volume and 90‐day poor outcome in patients with AIS and exerted a mediating effect of 15% to 30%.

The judgment of ischemic penumbra volume in this paper differs from previous literature findings, which suggest that the larger the ischemic penumbra volume, the better the prognosis, and this finding has some limitations. Based on the analysis in this study, for most participants, the larger the ischemic penumbra volume, the more extensive the neuronal cell damage and the more brain tissue that needs to be salvaged. When neuronal cells in the ischemic penumbra are subjected to sustained and extensive severe inflammatory responses and oxidative stress, the likelihood of neural repair in patients with AIS is reduced, and the worse the prognostic outcome is, the more the baseline ischemic penumbra volume is positively correlated with the poor outcome of prognosis. However, we found a mismatch between ischemic penumbra volume and prognostic outcome in a small number of participants (i.e., a small baseline mismatch volume with a poor prognosis and a large baseline mismatch volume with a good prognosis). This phenomenon may be related to the abundance of cerebral collateral circulation and the severity of the inflammatory response through in‐depth analysis. On the one hand, the rich collateral circulation of brain tissue provides more possibilities for ischemic tissue reperfusion. On the other hand, the inflammatory response after injury exerts a greater influence on the prognostic outcome. Numerous studies have shown that a series of inflammatory responses after stroke can accelerate neuronal death and lead to rapid loss of function (Shichita et al. 2012; Muhammad et al. 2021; Dirnagl et al. 1999). The exact mechanism is currently unclear, but a large number of hypotheses and studies suggest that the pathological changes after AIS are closely associated with severe immunosuppression (Chamorro et al. 2012; Magnus et al. 2012; Haeusler et al. 2008; Klehmet et al. 2009). NLR and MLR may reflect the balance between innate, adaptive, and inflammatory immune responses (Zuo et al. 2025). Following a stroke, peripheral immune cells are activated, leading to an increase in neutrophils, which release reactive oxygen species and proteases, thereby damaging the blood‐brain barrier and exacerbating the extent of brain injury. Meanwhile, apoptosis results in lymphocyte depletion, increasing the risk of infection and leading to worsening neurological function (Chen et al. 2022). Monocytes proliferate and activate in response to cytokine stimulation at the onset of injury, subsequently differentiating into macrophages. While clearing damaged cellular debris, they release cytokines that exacerbate the inflammatory response (Zhang et al. 2024). Some studies have speculated (Gao et al. 2021) that in AIS patients at the onset of the disease, platelet consumption occurs due to thrombus formation. Additionally, platelets interact with neutrophils after a stroke, and platelets can mediate neutrophil‐induced inflammatory responses (Kollikowski et al. 2020; Zhang et al. 2024). Therefore, a lower PNR can predict a poorer prognosis.

The inflammatory markers NLR and MLR may reflect the balance of innate, adaptive, and inflammatory immune responses. Platelets interact with neutrophils after stroke, and platelets can mediate neutrophil‐induced inflammatory responses (Zuo et al. 2025; Chen et al. 2022). It has also been found that NLR, MLR, and PNR levels are associated with 90‐day functional prognosis and risk of death (Cao et al. 2024; Geng et al. 2023; Zawiah et al. 2023; Liu et al. 2025). In addition to this, cytokines in the inflammatory response, such as tumor necrosis factor alpha and interleukins, have been found to influence the formation of neovascularization and collateral circulation, which in turn affects the recovery of brain tissue in the ischemic penumbra region (Zvejniece et al. 2020). These findings further emphasize the importance of focusing on the prognostic impact of the inflammatory response in the diagnosis and treatment of AIS, as well as the importance of aggressive anti‐inflammatory therapy in improving neural repair (Gu et al. 2022; Jin et al. 2023). Thus, baseline mismatch volume combined with inflammatory markers can better predict the prognostic outcome of AIS patients.

In recent years, MRI has become increasingly sophisticated, and its “perfusion‐diffusion mismatch” study has been widely used in clinical practice (Campbell et al. 2012). However, for time‐related diseases such as AIS, CTP is superior to MRI in terms of speed and accessibility (Smith et al. 2019). Therefore, CTP has been increasingly used by clinical emergency physicians to assess the prognosis of patients with AIS and to guide reperfusion therapy.

Furthermore, a multicenter randomized trial conducted by Zeinhom et al. (2024) confirmed that baseline NIHSS score and blood glucose level are independent predictors of poor prognosis in patients treated with alteplase (Zeinhom et al. 2025). This is consistent with the findings of the present study regarding the prognostic effects of elevated blood glucose level on admission and high baseline NIHSS score. Additionally, their research focusing on atrial fibrillation‐related embolic stroke in the Middle East and North Africa region revealed that medication adherence and the control of underlying diseases influence long‐term prognosis, which also aligns with the conclusions of this study (Zeinhom et al. 2024). Previous studies have verified that elevated blood glucose levels can exacerbate cellular damage in the ischemic penumbra (Anderson et al. 1999), while higher NIHSS and mRS scores are typically indicative of a larger infarct volume. With the expansion of the core infarct volume and the further aggravation of cellular damage in the ischemic penumbra, the neurological function recovery process of patients with AIS is directly impaired, ultimately resulting in poor outcomes.

There are some limitations of this study. First, the mechanisms of nerve repair after stroke are not clear. For example, the ischemic penumbra is a dynamic region whose size is related to the duration of cerebral ischemia, the compensatory capacity of collateral circulation, risk factors for cerebrovascular disease, and stroke complications. This study included AIS patients within 5 days of onset, which is a relatively long time frame. Some patients may have experienced blood flow reperfusion due to good collateral circulation, so CTP may have overestimated the volume of the ischemic penumbra. However, this study combined the effects of inflammatory markers on the ischemic penumbra to more accurately predict the prognosis. Second, due to interindividual differences in intracranial microvascular distribution and lesion extent, CTP may be falsely positive for low white matter flow and exaggerate core infarct size. However, this limitation will diminish with the widespread use of CT scanners covering the whole brain. Due to the existence of objective reasons, this study lacked the calculation of the blood pressure trend index within the first few hours of onset. The absence of this index may lead to an incomplete assessment of the association between blood pressure and prognosis, especially potentially missing the independent impact of dynamic blood pressure changes on outcomes. Finally, this study was a single‐center retrospective study with a small sample size and a single source of data, which did not take into account the variability between different regions and populations, which may affect the generalizability of the findings. In the future, we will conduct a multicenter prospective study to further validate the results of this study.

## 5 Conclusion

This study demonstrated that the larger the baseline mismatch volume, the worse the 90‐day prognosis of AIS patients. NLR, MLR, and PNR mediated the association between baseline mismatch volume and poor outcome at 90 days in AIS patients. In a small proportion of patients with AIS, baseline mismatch volume in combination with inflammatory markers may better predict prognostic outcomes. These findings suggest that emergency clinicians can utilize baseline mismatch volume to rapidly assess disease severity and guide aggressive therapeutic regimens, including endovascular and anti‐inflammatory therapies, to improve neurological function and long‐term quality of life in patients with AIS.

## Author Contributions

**Lianhong Ji**: data curation, formal analysis, investigation, methodology, visualization, writing – original draft. **Xinyu Xu**: data curation, formal analysis, investigation, methodology, visualization, writing – original draft. **Peian Liu**: formal analysis, investigation, methodology, visualization, writing – original draft. **Li Li**: formal analysis, investigation, methodology, visualization, writing – original draft. **Jiale Gan**: formal analysis, investigation. **Junqi Liao**: formal analysis, investigation. **Yongxing Deng**: data curation, formal analysis. **Hui Jiang**: data curation, formal analysis. **Yunfei Han**: data curation, formal analysis. **Wenlei Li**: conceptualization, data curation, funding acquisition. **Yuan Zhu**: conceptualization, funding acquisition, methodology, project administration, writing – original draft, writing – review and editing. **Minghua Wu**: conceptualization, funding acquisition, methodology, project administration, writing – original draft, writing – review and editing.

## Funding

This research was supported by the National Natural Science Foundation of China (Grant Nos. 82274428, 82474435), the Natural Science Foundation of Jiangsu Province (Grant No. BK20241996), the Administration of Traditional Chinese Medicine of Jiangsu Province (Grant No. ZT202102).

## Ethics Statement

The Ethics Committee of the Affiliated Hospital of Nanjing University of Chinese Medicine approved the clinical study, and the ethics approval document No. 2022NL‐010‐02 was implemented. This is a retrospective study, no written informed consent was required, all patient data were anonymized before analysis to ensure confidentiality, and no personally identifiable information was involved in the study.

## Supporting information




**Supplementary Information**: brb371219‐sup‐0001‐SuppMat.docx

## Data Availability

The data that support the findings of this study are available from the corresponding author upon reasonable request.
