# Supplementary material for: Inflammatory Markers Mediate the Prognosis of Baseline Mismatch Volume and 90‐Day Outcomes in Acute Ischemic Stroke Patients
Source: Brain Behav. 2026 Jan 19;16(1):e71219. doi: 10.1002/brb3.71219 (PMC12816761; doi:10.1002/brb3.71219)
Supplement: Supplementary file 1 — Supplementary Information: brb371219‐sup‐0001‐SuppMat.docx [file BRB3-16-e71219-s001.docx]

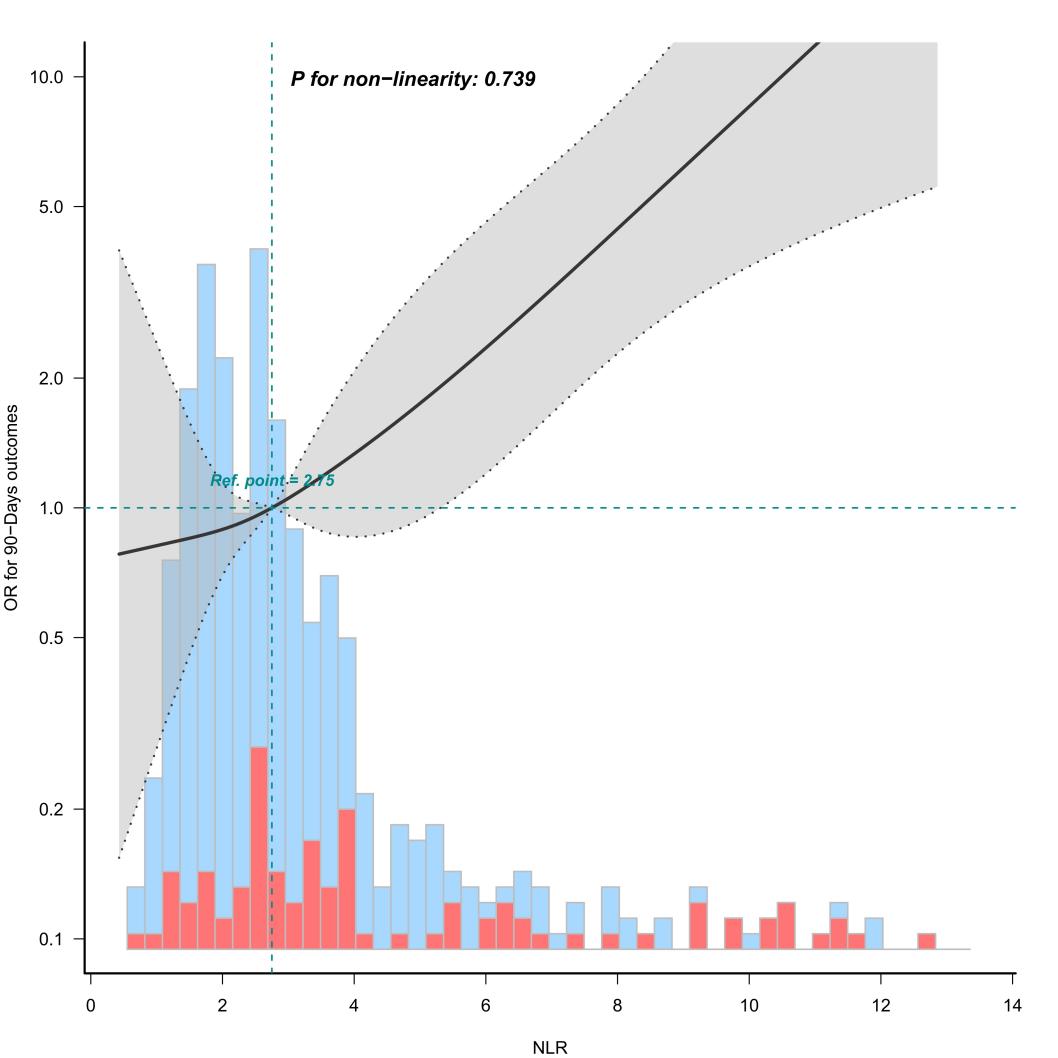


**Figure S1** Restricted Cubic Splines of the relationship between NLR and 90-day poor outcomes in AIS patients.


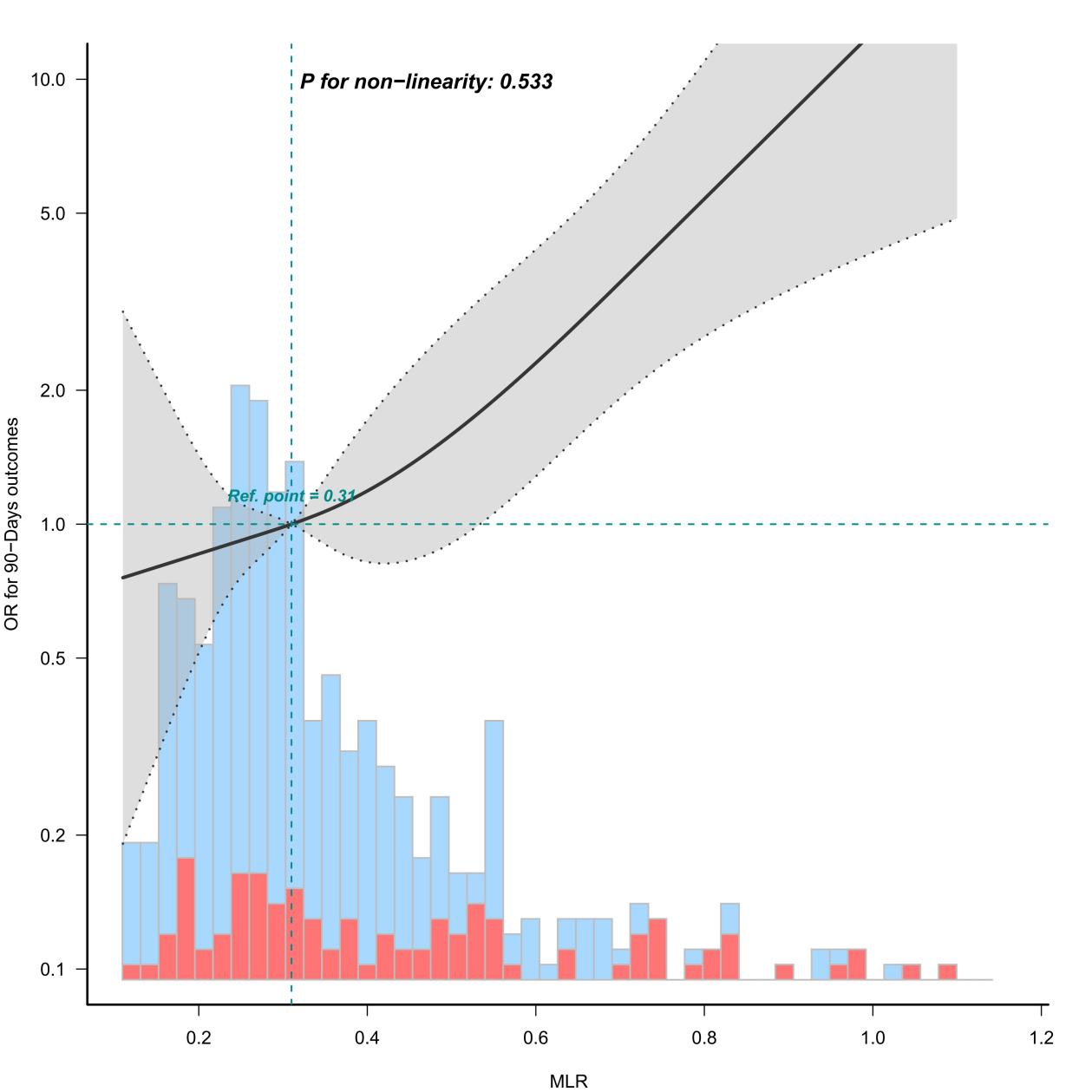


**Figure S2** Restricted Cubic Splines of the relationship between MLR and 90-day poor outcomes in AIS patients.


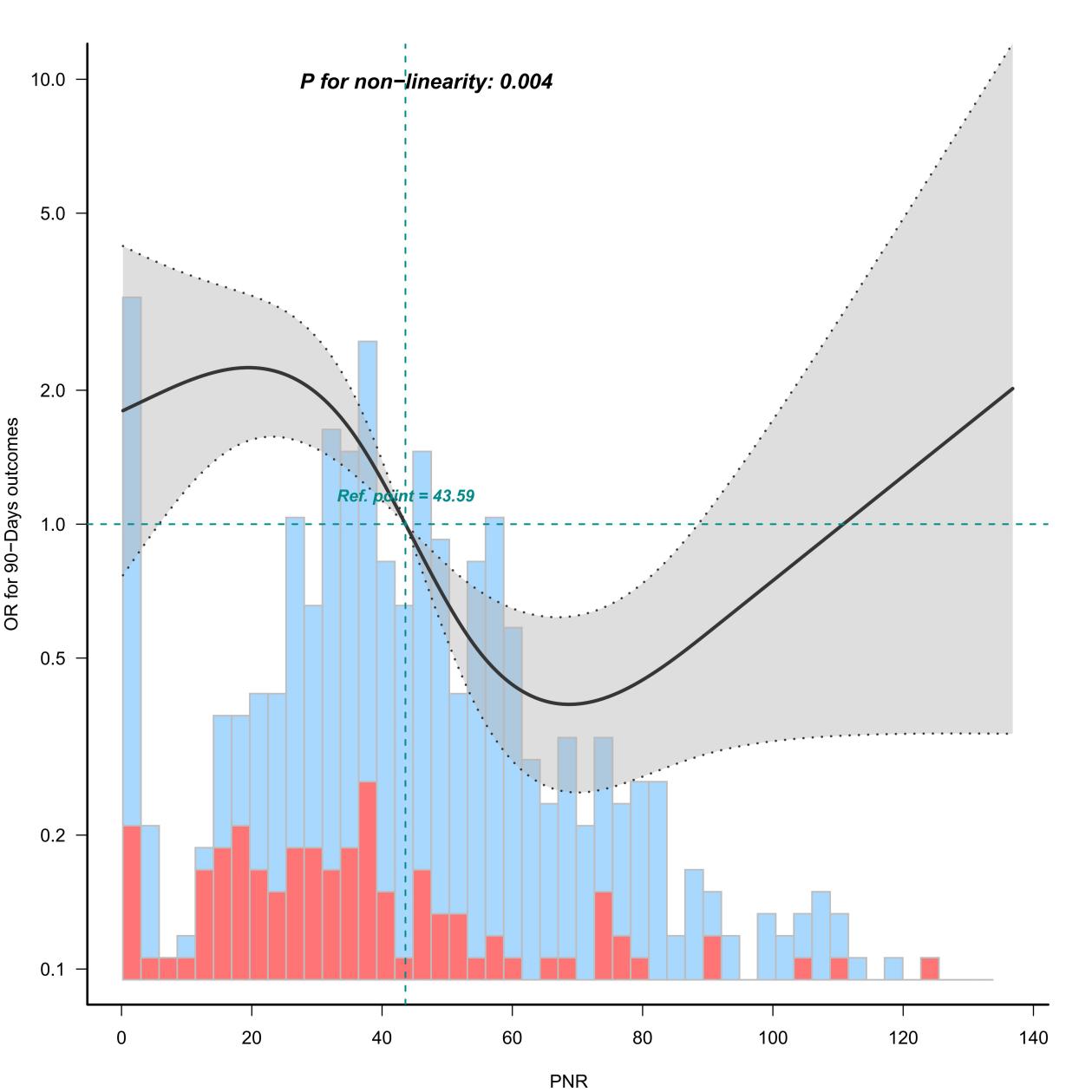


**Figure S3** Restricted Cubic Splines of the relationship between PNR and 90-day poor outcomes in AIS patients.

| **Table S1** Univariate and multivariate logistic regression analysis of NLR, MLR, PNR, and 90-day outcomes in patients with AIS. | | | | | | | | |
| --- | --- | --- | --- | --- | --- | --- | --- | --- |
| Inflammatory indexes | Model 1 | |  | Model 2 | |  | Model 3 | |
|  | OR (95% CI) | P‑value |  | OR (95% CI) | P‑value |  | OR (95% CI) | P‑value |
| NLR | 1.21 (1.13~1.3) | <0.001 |  | 1.21 (1.13~1.29) | <0.001 |  | 1.22 (1.14~1.31) | <0.001 |
| MLR | 1.73 (1.41~2.14) | <0.001 |  | 1.68 (1.36~2.07) | <0.001 |  | 1.71 (1.38~2.12) | <0.001 |
| PNR | 0.98 (0.97~0.99) | <0.001 |  | 0.98 (0.97~0.99) | <0.001 |  | 0.98 (0.97~0.99) | <0.001 |
| Model 1 was a univariate analysis. Model 2 was adjusted for gender and age.Model 3 was adjusted for gender, age, previous medical history, and medication history (OR: odds ratio; CI: confidence interval). | | | | | | | | |
